# Overloaded! Critical revision and a new conceptual approach for snow indicators in ski tourism

**DOI:** 10.1007/s00484-020-01867-3

**Published:** 2020-02-04

**Authors:** B. Abegg, S. Morin, O. C. Demiroglu, H. François, M. Rothleitner, U. Strasser

**Affiliations:** 1 Institute for Systemic Management and Public Governance, University of St. Gallen, 9000 St. Gallen, Switzerland; 2 Department of Geography, University of Innsbruck, 6020 Innsbruck, Austria; 3 Univ. Grenoble Alpes, Université de Toulouse, Météo-France Grenoble, CNRS, CNRM, Centre d’Etudes de la Neige, 38000 Grenoble, France; 4 Department of Geography and Arctic Research Centre, Umeå University (ARCUM), 901 87 Umeå, Sweden; 5 Univ. Grenoble Alpes, INRAE, LESSEM, 38000 Grenoble, France; 6 Schneezentrum Tirol am MCI, The Entrepreneurial School, 6020 Innsbruck, Austria

**Keywords:** Snow indicators, Ski tourism, Climate variability and change, Stakeholder process

## Abstract

Indicators are widely used in climate variability and climate change assessments to simplify the tracking of complex processes and phenomena in the state of the environment. Apart from the climatic criteria, the snow indicators in ski tourism have been increasingly extended with elements that relate to the technical, operational, and commercial aspects of ski tourism. These non-natural influencing factors have gained in importance in comparison with the natural environmental conditions but are more difficult to comprehend in time and space, resulting in limited explanatory power of the related indicators when applied for larger/longer scale assessments. We review the existing indicator approaches to derive quantitative measures for the snow conditions in ski areas, to formulate the criteria that the indicators should fulfill, and to provide a list of indicators with their technical specifications which can be used in snow condition assessments for ski tourism. For the use of these indicators, a three-step procedure consisting of definition, application, and interpretation is suggested. We also provide recommendations for the design of indicator-based assessments of climate change effects on ski tourism. Thereby, we highlight the importance of extensive stakeholder involvement to allow for real-world relevance of the achieved results.

## Introduction

The snow cover is characterized by a significant year-to-year variability which affects all relevant snow variables such as snow water equivalent (SWE), snow depth, and spatial and temporal snow coverage (Beniston et al. 2018). For the description of these basic variables, many statistical measures were developed, and thereby, the definition of indicators helps to simplify the complexity of the snow processes. According to their particular needs, many scientific disciplines provide individual definitions for indicators (e.g., Heink and Kowarik 2010). Here, we refer to a general definition: An indicator is used to simplify a complex reality and, more specifically, to “communicate information on environmental status and problems, to support response mechanisms and to monitor effects of these responses” (Smeets and Weterings 1999: 5). Hence, the snow indicators that are investigated in this paper are measures to track the snow state of the environment with respect to downhill skiing. They describe the snow conditions, variable both in space and time, at the regional scale and for time horizons from years to decades, be it the natural snow, or man-made machine snow—further on referred to as “technical snow”—that is deployed and groomed on the slopes of a ski area during the operating season.

Since the global phenomenon of climate change became documented by the first assessment report of the IPCC (1992), snow indicators have been increasingly used to assess the effect of climate change on the snow conditions, including the skiing conditions on the slopes of ski areas (see Steiger et al. 2019 for an overview). On the one hand, these investigations were of scientific interest. On the other hand, they were intended to support planning and decision-making in the ski tourism market at both the entrepreneurial and political levels (Abegg et al. 2007). However, it must be clearly stated that the climate change signal in the snow cover shows a small trend compared with its year-to-year variability (Marke et al. 2015). Hence, climate change effects are determinable only in long-term assessments (i.e., for several decades), whereas the interest of local ski tourism actors typically lies in the range of one (e.g., optimized snow management for the current season) to approximately 20 years (e.g., amortization period) (Abegg et al. 2017).

About the time when the IPCC published its first seminal reports, a profound and worldwide change took place in the operation of ski areas: the significant growth of snowmaking (e.g., Scott and McBoyle 2007 for North America and Spandre et al. 2015 for France). Technical snow allows to cope with the lack of natural snow and facilitates the grooming of the ski slopes. Resoundingly successful, an ever-increasing number of ski areas were equipped with snow guns, lances, pipe systems, pumping stations, and water reservoirs. Today, snowmaking (or sophisticated snow management in general) is an important part of the business. It helps to cope with “non-controllable” factors such as natural snowfall. Even more, it has become an indispensable planning and management tool to decouple skiing—at least partly—from the natural winter conditions. Or in the words of the CEO from an Austrian ski area: “In fact we don’t need snow, we make snow. Too much natural snow is bad for our business, because it means higher costs for slope grooming. Skiers only complain about natural snow pistes. They want smooth slopes that we can only provide with the help of machine-made snow. It sounds absurd, but the best scenario for us is less natural snow, low temperatures for snow production and lots of sun” (Trawöger 2014: 345). As a result of this development, operational aspects of ski tourism (such as ski season length or profitability) cannot be explained solely by climatic/meteorological (snow-related) variables.

In parallel, researchers have tried to catch up with the technical and commercial evolution of ski tourism and increasingly considered non-natural framing conditions for the snow and skiing conditions in their indicator definitions. Firstly, to account for operational and financial aspects of ski tourism, thresholds and ratings (and therefore a normative meaning) have been added to existing snow indicators. The most prominent example is probably the so-called 100-day rule. Bürki (2000: 42), for example, defined the 100-day rule as follows: “A ski resort can be considered snow-reliable if, in 7 out of 10 winters, a sufficient snow covering of at least 30 to 50 cm is available for ski sport on at least 100 days between December 1 and April 15.” Although widely accepted in both science and industry, the 100-day rule (and any other similar rule) is only an approximation of the required ski season lengths, and in contrast to how the rule was originally coined (Witmer 1986), the 100-day rule cannot be considered as a robust economic indicator because economic failure/success is dependent on much more than a simple number of days with a sufficient amount of snow (Abegg 2012). Secondly, to integrate technical snow means to define the snowmaking process. Consequently, the models used to calculate the snow indicators were extended with particular capabilities to simulate the production and grooming of technical snow, requiring a set of assumptions for the snow management strategy behind (Hanzer et al. 2018). Snowmaking, however, is complex (with many innovations potentially yet to come) and dependent on a series of physical, technical, operational, financial, and legal aspects that define the snow management process in real-world ski areas.

Over time, many indicators developed into “hybrids” (Heink and Kowarik 2010), combining meteorological/climatic (depending on the temporal horizon of their application, not further distinguished from hereon) with technical, operational, and financial aspects of ski tourism. Further, they became “overloaded” with regard to the meanings that have been attributed to these indicators—meanings that go way beyond of what can be stated with indicators based on physical (e.g., snow) conditions only. This, basically, applies to all indicators relying on pre-defined thresholds (be it a required ski season length or a given altitudinal line of snow reliability) and standardized snowmaking rules. These indicators may provide a coarse overview and can be used for general sensitivity analyses but, at the same time, they are prone to mis-/overinterpretation, and consequently even misinformation (e.g., notoriously misleading media headlines and over-simplified winner-and-loser discussions) (Scott et al. 2012: 210f). Actually, the aspects packed into these indicators are very different in nature: the climatic conditions—and, depending on those, the snow conditions—can be measured and more or less well projected by climate models, and they are representative for areas of regional or even larger scales (Gobiet et al. 2014). The technical, operational, financial, etc. framing conditions of a particular ski area, on the other hand, are hardly predictable (e.g., amount of available water, number of snow guns, or subsidies to buy/run the snow guns) and are, if assumed, only valid at the scale of a single ski area (Hanzer et al. 2014). Hence, the explanatory power of such hybrid indicators is limited: in their use origins a scale problem, a predictability problem, an arbitrariness problem, and an interpretation problem. Again, attempts to relate the effect of a regionally changing climate to particular local settings are prone to misinterpretation by practitioners and the media, especially in cases where the local settings strongly deviate from the assumptions made for the regional scale. To account for the uncertainties related to these assumptions, stakeholders can be involved to provide their local knowledge of the particular framing conditions in their region (Strasser et al. 2014).

In this paper, we provide an overview of what types of indicators have been developed and applied, we introduce a conceptual approach for the use of snow indicators and suggest a set of “de-loaded” indicators to be used for an unambiguous interpretation of the regional patterns of weather and climate variability effects. These basic indicators are not entirely new but mostly stripped from additional normative meanings. When applying the indicators, we suggest to distinguish between small-scale (e.g., individual ski areas) and large-scale (e.g., general policy and governance orientation) assessments. We further endorse a transdisciplinary research setting with local “case studies” and intensive stakeholder work to elaborate on both regional patterns of climate variability effects on ski tourism, and the very particular local consequences of these effects for particular ski areas. The cooperation with local stakeholder groups also holds the potential to be further developed into learning alliances to jointly elaborate on decision support and adaptation strategies.

This opinion paper is based on our long-time experience originating from research in a number of countries and cooperation with many partners. Many scientific colleagues and tourism stakeholders have contributed by conducting their own work, sharing their ideas/experiences and giving valuable feedback. The idea to write this article has evolved while being engaged in several third-party funded projects (listed in the acknowledgements). These projects focus on the impact of climate variability and change on the ski tourism industry and aim at providing decision support for ski tourism stakeholders. Key to all projects has been (and still is) the co-development of knowledge with stakeholders.

## Overview of existing snow indicators

The most common snow indicator for ski tourism is the number of days with a certain amount of snow on the ground, the “snow days.” Different thresholds have been proposed, for example 1, 5, 15, 30, or 50 cm. The snow depth requirements are dependent on slope characteristics: the rougher the terrain, the more snow is needed. Most ski areas, however, operate on landscaped terrain, i.e., slopes have been technically altered to reduce the required snow depth and to facilitate grooming. Therefore, 30 cm is typically taken for ungroomed snow—a threshold widely accepted since its introduction by Eckel (1938 in Witmer 1986) in both the science and ski tourism communities. The lower thresholds (e.g., 5 cm) are used for the “white winter landscape” (Strasser et al. 2014) or the “wintry atmosphere” (Schmucki et al. 2017)—an indicator that is not directly related to the operation of a ski area but to the look and feel of the environment.

In some studies (e.g., Scott et al. 2006), a day with ≥ 30 cm of snow is called a “skiable day,” and the number of days with snow depths ≥ 30 cm the “ski season length” (also: number of days between the ski opening (Snow Water Equivalent (SWE) > 120 mm w.e. (kg m^−2^) after 1 November for at least 5 consecutive days) and the ski closing (SWE < 80 mm w.e. for at least 10 consecutive days between the opening and 30 April), Marke et al. 2015). Based on the operational practices of ski areas in Eastern Canada, Scott et al. (2003, 2006, 2007) suggested that ski areas were assumed to close if one of the following conditions occurred: snow depth less than 30 cm, maximum temperature higher than 15 °C, or 2-day liquid precipitation exceeding 20 mm. The same authors also point to the difficulty to determine the length of the ski season with meteorological criteria only. Ski area managers may start the operation early, i.e., before the desired snow depth is reached, because they have to compete with neighboring ski areas (or to cash in during high-demand periods), and they may end it early because the demand is lacking and costs can be reduced (Scott et al. 2007). This is often the case towards spring when people prefer summertime activities although there is still a lot of snow in the ski areas (Mayer et al. 2018).

Starting in the late 1970s, a special meaning was attributed to a particular number of snow days. Referring to French ski areas, Barbier (1978) wrote that 120 snow days are necessary to assure the profitability of ski area investments. Similarly, Witmer (1986: 193) stated that “in order to have an economically viable investment in [Swiss] winter sports areas, among others, the installed facilities need to be utilized for at least 100 days per season, which is only possible with a snow cover of sufficient thickness.” In Australia, “60–70 days is about the minimum for a viable downhill ski operation” (Galloway 1988: 428), and in North America, Mieczkowski (1990: 254) proposed that at least 100 (in the East) or 120 days (in the West) are necessary “to be commercially viable.” Witmer’s suggestion, commonly known as the 100-day rule, has become very prominent. Applications include, for example, Abegg (1996), König and Abegg (1997), Elsasser and Bürki (2002), Durand et al. (2009), Steiger and Stötter (2013), and Pons et al. (2015) for Europe, Bark et al. (2010) and Dawson and Scott (2010, 2013) for North America, and Hendrikx and Hreinsson (2012) for New Zealand. Scholars looking at Australian ski areas still refer to Galloway (e.g., Pickering 2011). However, the 100-day rule (or any other similar rule) cannot be considered a robust economic indicator (Abegg 1996, 2012). Economic success is dependent on sufficient snow at the right time (e.g., key periods), good weekend-weather, and many other non-climate-related factors (e.g., Elsasser and Bürki 2002). This has been known for long, but still, the 100-day rule is utilized as a proxy for the profitability, viability, and even sustainability of ski areas.

The “line of snow reliability,” combining the 100-day rule with altitude, goes back to earlier research in Switzerland. Föhn (1990) found that the criteria for the 100-day rule are met in areas above 1200 m. This line of snow reliability, he further suggested, will move up to 1500 m assuming a + 3 °C warming. Messerli (1990) used this information to draw a map, identifying a critical zone between 800 and 1500 m with ski areas being “at risk” or “highly at risk.” Abegg (1996) adapted Föhn’s approach and calculated the number of naturally snow-reliable ski areas under current and future climate conditions: a ski area was considered naturally snow reliable if the upper half of its altitudinal range was located above the respective lines of snow reliability. The same approach with varying baselines for natural snow reliability (i.e., lower in the Eastern part and higher in the Southern and South-Western part of the European Alps) was later applied in a much larger study for the OECD (Abegg et al. 2007). It was suggested to use the line of snow reliability to detect patterns in the geographical distribution of naturally snow-reliable ski areas. Snow conditions, however, are highly variable over space, and a highly aggregated line of snow reliability does not account for the complexity of the snow processes in mountain terrain (Steiger 2010). Further, it was recommended to not make statements on individual ski areas (Abegg et al. 2007: 32). The line of snow reliability, however, can be easily misinterpreted as a simple tool to rate individual ski areas, i.e., to distinguish between “winner” and “loser.” Financial institutions, for example, used the line of snow reliability to check on the ski areas’ credit ratings (e.g., Elsasser and Bürki 2002; Scott et al. 2007).

The ski season, as most tourism seasons, can be divided into different periods (e.g., Scott et al. 2007; Spandre et al. 2016a). Some periods are economically more important than others—an example being the Christmas holidays for mid-latitude ski areas in the Northern hemisphere. Consequently, Scott et al. (2008: 579) introduced the Christmas–New Year’s indicator as the “probability of being operational during the economically critical Christmas–New Year’s holiday period”, thereby referring to ≥ 30 cm of snow from 22 December to 2 January. Steiger and Abegg (2013) adopted the Christmas indicator, extended the period by 2 days (22 December to 4 January) and added the season opening indicator (snow depth ≥ 30 cm on 8 December). In Austria, the public holiday on 8 December (the Christian holy day of the Immaculate Conception)—or the weekend closest to this date—is the traditional opening day for many larger ski areas.

Many scholars use probability thresholds to rate the indicators. Wanner and Speck (1975) give an early example stating that their snow reliability criterion (≥ 90 days with ≥ 30 cm of snow) must be met in 90% of the winters. Often, several thresholds are available. It was suggested, for example, that the 100-day rule must be fulfilled in 70% (Abegg 1996) or 90% (Steiger and Mayer 2008) of the winters. Scott et al. (2008) suggested a 75% threshold for the Christmas–New Year’s indicator, and Steiger and Abegg (2013) who combined the 100-day rule, the Christmas indicator, and the season opening indicator used a 70% threshold for all three indicators. Steiger and Stötter (2013) went a step further and defined a “snow reliability classification scheme” considering the 100-day rule, the Christmas indicator, and the share of open skiing terrain. Probability thresholds (≥ 80%, 50–79%, and < 50%) were attributed to the Christmas indicator and the share of open skiing terrain resulting in a classification scheme ranging from “excellent” to “very poor.” There is a reasoning behind these thresholds: the indicators must not be fulfilled every year because of the changing quality of the snow season (“good versus bad snow years”) and the assumed ability of the ski areas to cope with a limited number of “bad” seasons and/or to compensate bad with good seasons. Why specific thresholds were chosen, however, often remains unclear. Some scholars (e.g., Abegg 1996; Steiger and Stötter 2013) double-checked with ski area operators, others did not—in general, information about stakeholder involvement is scarce.

Generally, snow days, season lengths, the 100-day rule, etc. can be calculated for both natural and technical snow. Snowmaking, however, shifts the perspective from natural conditions to more technical, infrastructural, and operational aspects. Scott et al. (2003) introduced a ski season and snowmaking simulation model (SkiSim). Snowmaking within SkiSim is based on technical capacities and operational practices which were derived from communication with ski industry stakeholders in Eastern Canada and include the start and the end date of snowmaking (22 November to 30 March), the snow base to maintain until 30 March (60 cm), the temperature required to start snowmaking (− 5 °C), and the snowmaking capacity (10 cm/day) (see also Scott et al. 2006, 2007). It is important to add that Scott et al. (2008: 583f) stated that “in order to compare the relative impact of projected climate change …, a single hypothetical ski area with identical characteristics (e.g., size, snowmaking capacities, and practices) was modelled at each study area. This approach isolates the importance of climate and projected climate change at each location, rather than assessing the relative technological (e.g., snowmaking) and business (e.g., four season operation) advantages of individual ski areas.” The SkiSim model was further developed (SkiSim 2.0) with refined snowmaking rules to better represent operational decisions over the ski season, i.e., distinction between base-layer snowmaking at the beginning of the season and reinforcement snowmaking later in the season (Steiger 2010). Applications in the European Alps with slightly adjusted snowmaking rules (e.g., snowmaking window from 1 November to 31 March instead of 22 November to 30 March) include Steiger and Abegg (2013) and Steiger and Stötter (2013). Pons et al. (2015) used a similar approach in the Pyrenees. Hanzer et al. (2014) developed a detailed, physically based model of technical snow production taking into account the ambient conditions and available snowmaking infrastructure. The model explicitly considers the topography and geometry of existing ski slopes, applies pre-defined snowmaking practices (i.e., base-layer and reinforcement snowmaking periods), incorporates grooming and the skier-induced downward transport of snow on the slopes, and tracks the water and energy consumption of the snow machines. In Marke et al. (2015), this model was applied to several ski areas in the Schladming region (Austria). Spandre et al. (2016b) implemented snow grooming and snowmaking in the physically based, multilayer snowpack model Crocus (Vionnet et al. 2012). This model was used to analyze past and future snow conditions in French ski resorts (Spandre et al. 2019a, b), both using the reliability line approach, and in an implementation where the spatial structure of each ski resort in a given region (French Alps) was explicitly represented. Hanzer et al. (2018) summarized the explicit implementation of snow grooming and snowmaking in the snow models AMUNDSEN (Strasser 2008; Hanzer et al. 2014; Strasser et al. 2019), Crocus (Vionnet et al. 2012; Spandre et al. 2016b), and SNOWPACK/Alpine3D (Bartelt and Lehning 2002). In summary, scholars have developed various, i.e., more or less standardized, ways to incorporate snowmaking into their snow models. It is interesting though how they deal with uncertainties. In most cases (at least in more recent studies), scholars take into account climate model ensembles and different emission scenarios but still rely on one single set of assumptions to define the snowmaking capacities and practices.

Other scholars focused on the snowmaking potential. Rixen et al. (2011), for example, calculated the number of potential snowmaking days, i.e., days with a dew point temperature ≤ − 4 °C; Hartl et al. (2018) did the same for German and Austrian weather stations, using a mean daily wet-bulb temperature threshold of − 2 °C, and computed the significance of historical trends. Hennessy et al. (2008) modelled potential snowmaking hours under current and future climate conditions in Australia, again using a − 2 °C wet-bulb temperature threshold. Similarly, Hendrikx and Hreinsson (2012) modelled potential snowmaking hours in New Zealand. They noted that a threshold of ≤ − 3 °C wet-bulb temperature is typically used in operational settings. However, they used a higher threshold of ≤ − 1.7 °C as discussions with the ski area operators indicated “that in warm years this was how the machines were operated, despite the cost and small amounts of snow that can be made at these warmer temperatures.” Spandre et al. (2015) analyzed past conditions of snowmaking hours in the French Alps and discussed the impact of various wet-bulb temperature thresholds.

Beyond the snow depth and duration indicators, additional climate variables play complementary roles in assessing the skiing conditions. Berghammer and Schmude (2014), for example, introduced the “Optimal Ski Day” as a day with no precipitation, a perceived temperature between − 5 and + 5 °C, more than 5 h of sunshine, and a wind speed less than 10 m/s, in addition to a minimum snow depth of 30 cm on the slopes and a white scenery in the surroundings. Demiroglu et al. (2018) identified the “ideal summer skiing day” through a consumer survey in Norway as one with no wind, an open sky, relatively warm temperature (10 to 20 °C), and wet or “corn” snow quality. Likewise, the China Meteorological Administration (2017) developed the “Meteorological Index of Skiing” by taking into account wind, temperature, and precipitation. Based on these antecedents and industrial inputs, a comprehensive climate index for ski tourism is proposed by Demiroglu et al. (2019). In this paper, however, we focus on the most essential component of ski tourism climatology—the snow cover—and do not provide an in-depth examination of individual or combined effects of the above-mentioned additional variables.

## Conceptual approach and list of indicators

The following conceptual base for the use of indicators in ski tourism has been developed and proven useful in the course of several third-party-funded research projects in Alpine ski resorts (listed in the acknowledgements). These projects focus on the impact of climate variability and change on the ski tourism industry. In all these projects, intensive stakeholder work led to the formulation of general requirements that the indicators ideally fulfill. These include the following:

(i) *Simplicity*. A good indicator comprises complex processes such that its meaning is easily understandable. This is the original purpose of an indicator.
(ii) *Transferability*. The indicator should be applicable independent of space and time.
(iii) *Universality*. The indicator should be independent of any particular condition.
(iv) *Neutrality*. The indicator itself should be free of any normative assignment.
(v) *Quantitative nature*. The indicator should be a numeral that can be measured or computed by means of a numerical procedure.
(vi) *Applicability in climate change impact studies*. The indicator should be calculable using variables that can be provided by coupled climate and snow models.
(vii) *Validity*. The indicator should be an accepted measure to assess real-world phenomena.
(viii) *Compatibility*. The indicator should be in line with the paradigms of transdisciplinary research (Lang et al. 2012).

### Development and use of indicators

**Fig. 1:**
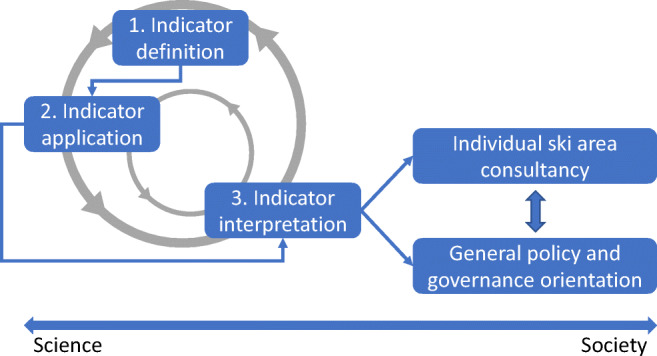
The three-step iterative procedure of indicator definition, application, and interpretation. The outcome of this transdisciplinary process can (i) be anchored to support individual ski areas (explicit approach with case studies) or (ii) be used to estimate regional patterns of ski tourism conditions with generalized assumptions for the local snow management practices (modified after Strasser et al. 2014)

It is suggested to distinguish between indicator definition, application, and interpretation (Fig. 1). All three steps are iterative in nature; whenever a set of indicators is defined and associated with value and scale, the interpretation of the results may be the origin of new questions regarding both indicator definition and application. Use of indicators is a transdisciplinary process and requires appropriate formats to ensure proper communication and cooperation between the groups participating in the process, e.g., stakeholder workshops. In the ideal case, it initiates a mutual learning alliance to jointly develop local decision support and adaptation strategies (Strasser et al. 2014).

#### Step 1

The indicator is defined. While indicator definition is often done by scientists alone, we suggest to co-operate with stakeholders from the very beginning. Ideally, the same stakeholders who later do the interpretation of the indicator values participate in the definition process. The co-definition of the indicators helps to better understand the investigated phenomenon and to come up with indicators relevant for both science and practice. During the definition process, neither scale and value nor a normative meaning is attributed to the indicator. It is (just) a conceptual construct for the complex processes and/or conditions of a certain phenomenon in the real world.

#### Step 2

The indicator is applied and gets a value and a scale, e.g., by means of an analysis of observation time series or a numerical simulation of the change of physical variables in time, calculated by the computer code of a (e.g., snow or hydrological) model for a given domain and time period. The scientists setting up the simulation runs and computing the indicator values are not necessarily the same as the ones who have contributed to the indicator definition (and/or selection). In this step, an interaction between researchers and stakeholders is not necessarily required, but it may be interesting for stakeholders to be part of this step. It may be iterative with step 1 (re-definition of an indicator after application). Still, no normative meaning is associated, neither to the indicator itself nor to its value: it is just a numeral.

#### Step 3

The indicator is interpreted. In this step, the indicator is turned into something specific, i.e., valid for a given time and space, and usually gets a normative meaning. This cannot not be done by scientists alone but is a co-operative procedure and requires stakeholder involvement. Often the interpretation of an indicator is the second part of a stakeholder process that started in the first step. The stakeholder may not be a single person, but a group of people, e.g., employees of a company or members of a political body. It is not a priori given that the members of the group agree on the normative meaning of the indicator value, and hence, divergent perceptions of the normative meaning of a certain indicator value may exist. The result of the interpretation process can be the basis for operational, economic, strategic, or political decisions (depending on the case). The third step may require iteration with step 1 (definition of a new additional indicator after evaluation of the original ones) and step 2 (communication of uncertainties in the method and its results).

The indicators can be used for both small- and large-scale assessments. If the indicators are coupled with the real-world framing conditions of a particular ski area—jointly formulated with local stakeholders—the interpretation may provide the basis for individual ski area consultancy. If the indicators are coupled with a set of generalized assumptions that define the framing conditions of a “standardized” ski area, the interpretation may provide the basis for general policy and governance orientation. Large-scale assessments based on a set of generalized assumptions also benefit from cooperation with ski tourism stakeholders. Stakeholder knowledge was already integrated in the past, although in a rather limited and non-transparent way. Further, only one set of assumptions was typically used to define the framing conditions of the ski areas—a limitation given the fact that the technical, operational, and financial settings of ski areas differ in many respect. To give an example, snowmaking rules are based on a series of assumptions including temperature thresholds and snowmaking capacities (cm/day). Often, only one set of assumptions is taken into consideration. However, these parameters vary, and changes in the definition of the parameters (e.g., higher threshold for the wet-bulb temperature and/or higher capacity of the snowmaking facility) may result in significantly different outcomes (e.g., a markedly higher amount of technically produced snow). For future research, we recommend (i) to extend stakeholder involvement, (ii) to use several sets of assumptions to define snowmaking capacities and practices, and (iii) to properly communicate all the assumptions and their consequences.

### List of indicators

Table 1 lists our set of proposed snow indicators, including name, a short description, the unit, and a remark on how to calculate the indicator. Snow depth thresholds, wet-bulb temperature thresholds, and time spans for key periods, base-layer, and reinforcement snowmaking are not pre-set and have to be defined in cooperation with stakeholders. Apart from the Christmas–New Year’s period mentioned in the table, additional key periods, usually related to school breaks or public holidays, can be defined. Incorporating technical snow means to define the factors that steer the snowmaking process as discussed in previous paragraphs. In further post-processing steps, the duration of the continuous snow season can be calculated by using the start and the end date of the snow season. If calculated for longer time periods (decades), statistical measures such as mean and standard deviation of the indicator values can be computed. In a specific research setting, the percentage of years meeting a certain threshold could be calculated as well: ski area operators, for example, might be interested in the percentage of years in which the daily snow depth thresholds over the economically critical Christmas–New Year’s period are fully met.

**Table 1:** List of snow indicators

No.	Name	Description	Unit	Snow type		Calculation/computation method
				Natural	Technical*	
1	White winter landscape	Number of days with at least *x* cm of snow on the ground	Number of days	✓		Count the number of days from 1 August of year *N* − 1 to 31 July of year *N* fulfilling the condition “snow depth ≥ *x* cm”
2	Snow days	Number of days with at least *x* cm of snow on the ground	Number of days	✓	✓	Count the number of days from 1 August of year *N* − 1 to 31 July of year *N* fulfilling the condition “snow depth ≥ *x* cm”
3a	Start of the snow season	First date of the longest continuous period with at least *x* cm of snow on the ground	Date	✓	✓	Identify the longest continuous period from 1 August of year *N −* 1 to 31 July of year *N* where snow depth is continuously above *x* cm. The first date, within this continuous period, meeting the condition “snow depth ≥ *x* cm”, is the start of the snow season. The last date, within this continuous period, meeting the condition “snow depth ≥ *x* cm”, is the end of the snow season
3b	End of the snow season	Last date of the longest continuous period with at least *x* cm of snow on the ground	Date	✓	✓	
4	Key period	Number of days with at least *x* cm of snow on the ground within economically critical key period(s)	Number of days	✓	✓	Define critical key period(s) (day 1 to day *x*) and count the number of days fulfilling the condition “snow depth ≥ *x* cm”
5a	Snowmaking potential for base-layer snowmaking	Number of hours with wet-bulb temperature lower than – *x* °C	Number of hours		✓	Define periods for base-layer and reinforcement snowmaking, compute wet-bulb temperature (*T*_WBT_) from temperature and relative humidity, and compute number of hours with “*T*_WBT_ ≤ − *x* °C” within the respective periods
5b	Snowmaking potential for reinforcement snowmaking	Number of hours with wet-bulb temperature lower than – *x* °C	Number of hours		✓	

*Depending on the specific snow model setting, “technical” may refer to “natural but groomed,” “natural and machine-made,” and “natural, machine-made, and groomed” snow

## Computation of the indicators

Apart from the proper definition (step 1) and application (step 2) of an indicator, its acceptance in the interpretation (step 3) significantly depends on the adequacy of the datasets and tools employed for its calculation. This has a strong influence on the level of confidence placed by all interested parties at the third stage of the process. Based on available resources and methods, the choice of the modelling framework used for the calculation of the indicators can also be discussed with stakeholders. Here we describe some key elements regarding the data and methods used for the calculation of the indicators.

### Geographical settings and time periods

While the target spatial domain of indicators used to assess the snow conditions in one or several ski areas comprises the ski slopes and the surrounding environment of the ski areas, observation and simulation data for a limited number of geographic locations can be employed. In situ meteorological and snow data from long-term observations are sometimes available at one or two locations in or around a given ski area. However, these meteorological and snow monitoring stations are often not situated at locations representative for snow conditions on ski slopes. Nevertheless, in situ time series have long been used for the assessment of both snow and climatic conditions at the local and regional scales. Beyond the use of in situ data for the calculation of indicators for each location, in situ data can be interpolated or extrapolated in space, in order to generate driving data for snowpack models spanning a wider range of locations within and around ski areas (e.g., Hanzer et al. 2014) and compute spatially distributed indicator values for entire ski areas (e.g., François et al. 2014; Spandre et al. 2019a). In contrast, mountain areas are generally inadequately represented in large-scale climate datasets, both due to a lack of some of the key processes in mountain regions in the models and to their too coarse spatial resolution (Hock et al. 2020). Approaches bringing together in situ observations, large-scale reanalysis, and climate model output hold potential in providing consistent estimates of meteorological and snow conditions in ski areas for past and future conditions (Durand et al. 2009; Marke et al. 2015, 2018). However, such combined products are more difficult to convey to non-experts and require further explanations to communicate their strengths and limitations.

Due to the high inter-annual variability of meteorological and snow conditions in mountain areas, multi-decadal time series are required to assess changes in snow conditions (Beniston et al. 2018; Hock et al. 2020). The time scales for climate change assessments exceed those of the evolution of the ski tourism industry, their business models, and management practices (see, e.g., the rapid development of snowmaking over less than two decades, compared with long-term snow cover changes; see Hock et al. 2020, and references therein). This must be taken into account for the interpretation of past and future changes of the snow indicators. Hence, for any assessment based on climate change projections (mid-twenty-first century or even later) only coarse assumptions for the future development of snow management infrastructure, amortization time scales, and the social, economic, and political drivers influencing the highly dynamic ski tourism market can be made. As a result, conclusions for specific ski areas can only be drawn from such predictions, bearing in mind these important considerations.

### Climate scenarios, uncertainties, and relevant variables

The assessment of future climate change relies on the use of climate change scenarios, consisting of global climate models (GCMs) fed by greenhouse gas emissions scenario (e.g., representative concentration pathways, RCP, Moss et al. 2010). Downscaling and adjustments are required prior to using global and regional climate models in mountain regions and in order to drive specific impact models such as snow models (e.g., Verfaillie et al. 2017). The future climate information should be provided and processed in a manner which makes it possible to assess the uncertainty attached to the indicators. Ensemble approaches, using several climate scenarios corresponding to various RCP, make it possible to quantify the variability and uncertainty affecting the results, and represent the results in a manner that is consistent with the natural and forced (due to anthropogenic greenhouse emission) variability of the mountain climate. These approaches also make it possible to compute future values of the indicators, from which mean values and frequency of occurrences (e.g., number of years when the indicators exceed a given threshold) can be computed (e.g., Steger et al. 2013; Verfaillie et al. 2018). Secondly, the downscaling and adjustment methods should provide the variables required for the computation of the indicators at the adequate time resolution. For example, not only daily temperature and precipitation data are required for snow indicator computations but also sub-diurnal values and a wider range of variables including liquid/solid precipitation partitioning, relative humidity, and wind speed, which govern snowmaking practices and are used in state-of-the-art models (e.g., Hanzer et al. 2014, 2018; Spandre et al. 2016b).

## Conclusions

In this paper, we elucidate that the use of indicators as measures to track the snow state of the environment with respect to downhill skiing deserves particular attention and care. We argue that operational aspects of ski tourism (such as its profitability) cannot be explained with climatic (snow-related) parameters. “Overloading” the natural environmental criteria in the definition of the indicators with regulation criteria originating from technical, operational, or commercial aspects of skiing will result in a loss of the explanatory power of the indicators, since these latter are specific for the local scale. Whereas the evolution of the climate follows physical principles and certain well-defined emission scenarios of greenhouse gases in more or less continuous trends, difficult though to project into the future, the technical, operational, and commercial framing conditions of a particular ski area can undergo step-like reorganizations which are not predictable; the technical infrastructure, the snow management practice, the financial situation, and the amount of water available are prominent examples for non-predictable, yet very specific and local driving factors. If not anchored with local stakeholder knowledge, but interpreted for a particular ski area, hybrid indicators induce a scale problem, a predictability problem, an arbitrariness problem, and an interpretation problem.

From a conceptual point of view, we have collected a list of general requirements to be ideally fulfilled by each indicator. It is suggested to split the indicator process into the three steps of indicator definition, application, and interpretation to deal with the above-mentioned problems. Normative attribution is not included in the indicator’s definition or calculation procedure but is part of the interpretation. This interpretation is individual and likely to vary from case to case (i.e., from person to person or from ski area to ski area etc.). The development and use of the indicators is therefore set up as a transdisciplinary and recursive process. Further, it is suggested to clearly distinguish between two ways of using the indicators: The first one refers to existing real-world ski areas and takes into account as many local peculiarities as possible to specify the framing conditions of the respective ski areas in a case-study type of assessment. The second way refers to a set of generalized assumptions to integrate the financial, operational, etc. framing conditions of ski tourism into the indicator definition.

Whereas the first approach provides the potential for direct consultancy of particular ski areas at the local scale, the latter approach is used to estimate the effect of a changing climate on several or many ski areas. By intention, this second method holds the potential for a comparative assessment of skiing conditions at regional scales, but the required assumptions that have to be specified to include the technical, operational, and commercial aspects of skiing into the indicator definition are difficult to transfer in space and time. The required generalization hence reduces the explanatory power and normative potential of the indicator when it comes to its interpretation at the local scale (i.e., for a particular ski area). This pitfall can be easily overseen in indicator-based ski tourism assessments. Yet, the more general and regionally applicable approach is a way to provide general policy and governance orientation for decision support and the development of adaptation strategies to cope with the effect of a varying climate. Recently, successful attempts were undertaken to further refine the snow management rules at the regional scale (Spandre et al. 2016a). Its usability mostly profits from scenario-type consideration of an ensemble of operation strategies, proper translation and communication of the methodology in the interpretation process, and documented interfaces to integrate local knowledge into the research process.

Both ways to use the indicators benefit from extensive stakeholder integration. For the first approach, stakeholder involvement is indispensable to specify the particular case, and for the second, it is paramount for the relevance of the achieved results. Recursive iterations in the co-production of knowledge across all three steps of the indicator process facilitate the development of relevant results and hold the risk of misunderstandings and misinterpretations at bay. Our approach is ready to be further developed and to be applied in different settings within ski tourism and beyond, i.e., in risk analysis, DPSIR (driving forces, pressures, states, impacts, and responses) approaches (Smeets and Weterings 1999), and scenario generation. Stakeholder involvement though is key to real-world significance.
